# Optimization and evaluation of a live virus SARS-CoV-2 neutralization assay

**DOI:** 10.1371/journal.pone.0272298

**Published:** 2022-07-28

**Authors:** Anders Frische, Patrick Terrence Brooks, Mikkel Gybel-Brask, Susanne Gjørup Sækmose, Bitten Aagaard Jensen, Susan Mikkelsen, Mie Topholm Bruun, Lasse Boding, Charlotta Polacek Strandh, Charlotte Sværke Jørgensen, Karen Angeliki Krogfelt, Anders Fomsgaard, Ria Lassauniere

**Affiliations:** 1 Department of Virus & Microbiological Special Diagnostics, Statens Serum Institut, Copenhagen, Denmark; 2 Department of Clinical Immunology, Copenhagen University Hospital, Copenhagen, Denmark; 3 Department of Clinical Immunology, Zealand University Hospital, Naestved, Denmark; 4 Department of Clinical Immunology, Aalborg University Hospital, Aalborg, Denmark; 5 Department of Clinical Immunology, Aarhus University Hospital, Aarhus, Denmark; 6 Department of Clinical Immunology, Odense University Hospital, Odense, Denmark; 7 Danish National Biobank, Statens Serum Institut, Copenhagen, Denmark; 8 Department of Molecular and Medicinal Biology, Institute for Science and Environment, Roskilde University, Roskilde, Denmark; 9 Infectious Diseases Unit, Clinical Institute, University of Southern Denmark, Odense, Denmark; Waseda University: Waseda Daigaku, JAPAN

## Abstract

Virus neutralization assays provide a means to quantitate functional antibody responses that block virus infection. These assays are instrumental in defining vaccine and therapeutic antibody potency, immune evasion by viral variants, and post-infection immunity. Here we describe the development, optimization and evaluation of a live virus microneutralization assay specific for severe acute respiratory syndrome coronavirus 2 (SARS-CoV-2). In this assay, SARS-CoV-2 clinical isolates are pre-incubated with serial diluted antibody and added to Vero E6 cells. Replicating virus is quantitated by enzyme-linked immunosorbent assay (ELISA) targeting the SARS-CoV-2 nucleocapsid protein and the standardized 50% virus inhibition titer calculated. We evaluated critical test parameters that include virus titration, assay linearity, number of cells, viral dose, incubation period post-inoculation, and normalization methods. Virus titration at 96 hours was determined optimal to account for different growth kinetics of clinical isolates. Nucleocapsid protein levels directly correlated with virus inoculum, with the strongest correlation at 24 hours post-inoculation. Variance was minimized by infecting a cell monolayer, rather than a cell suspension. Neutralization titers modestly decreased with increasing numbers of Vero E6 cells and virus amount. Application of two different normalization models effectively reduced the intermediate precision coefficient of variance to <16.5%. The SARS-CoV-2 microneutralization assay described and evaluated here is based on the influenza virus microneutralization assay described by WHO, and are proposed as a standard assay for comparing neutralization investigations.

## Introduction

Severe acute respiratory syndrome coronavirus 2 (SARS-CoV-2) antibodies induced through vaccination or infection can confer protection by blocking or neutralizing virus entry into the host cell by binding functional sites on the surface protein(s). Vaccine studies in nonhuman primates established that binding antibodies specific to the spike surface protein of SARS-CoV-2 and neutralizing antibodies are correlates of protection against upper and lower respiratory tract infection [1]. Quantitating neutralizing antibodies *in vitro* may therefore support i) the evaluation of vaccine-induced protective antibody responses [2–4]; ii) selection of convalescent plasma for clinical trials and compassionate care; iii) virus antigenic characterization [5–8]; and iv) defining immune evasion [9, 10]. SARS-CoV-2 is antigenically distinct from other β-coronaviruses known to infect humans and, thus, require novel methods to detect and quantitate antibodies specific for the virus.

Neutralization assays typically use live biological components that include actively replicating cells and viruses, in contrast to standard serological assays, for example enzyme-linked immunosorbent assay (ELISA), that use non-replicating assay components like protein antigens. As a result, neutralization assays are more subject to intra- and inter-assay variation. Variance beyond typical variability associated with standard ELISA is introduced by virus receptor expression level, cell density, cell morphology, virus infectiousness, and modulation of cell and virus chemistry during the process. An inter-assay variance of 20% is generally considered acceptable in standard immunoassay evaluation [11–14]. However, due to the complexity of the virus neutralization process and a neutralization assay, an inter- and intra-assay variation of 25–30% is deemed acceptable for cell-based assays types [15–17]. To minimize variance and ultimately achieve a robust and precise platform to reliably quantify neutralizing antibodies, it is important to optimize each variable in a neutralization assay.

We present an optimized and well characterized live SARS-CoV-2 microneutralization assay. The principle is based on the influenza virus neutralization assay described by the World Health Organization [18] which is used by influenza reference laboratories worldwide. The ability of the assay to attain reliable results were evaluated by addressing precision, linearity, and levels of detection and quantification. We furthermore provide novel powerful normalization tools that markedly reduce assay variation, thus offering enhanced assay standardization.

## Materials and methods

### Assay principle

Plasma or serum samples are titrated and incubated with a predetermined amount of virus for one hour to allow opsonization. The virus and sample suspension is subsequently transferred to a Vero E6 cell monolayer. In the presence of neutralizing antibodies, virus inoculum is blocked and the subsequent cell-infection reduced. The level of infection is measured in a semi-quantitative ELISA targeting the SARS-CoV-2 nucleocapsid protein. The level of nucleocapsid protein is directly proportional to the amount of virus inoculum and enables quantitation of virus inhibition in the presence of antibodies. A 50% inhibition cutoff is calculated from quadruplicate control wells containing virus without serum and mock infected cell controls. The last serum or plasma dilution inhibiting virus growth below this cutoff is reported as the 50% neutralization titer (here referred to as categorical 50% neutralization titers). Alternatively, a more precise titer is calculated by fitting all sample dilution determinations with a 4-parameter logistic regression curve and determining the intercept with cutoff. The full optimized protocol is available on www.protocols.io (DOI: doi.org/10.17504/protocols.io.q26g74ww3gwz/v2).

### Cells

Vero E6 cells (green monkey kidney cells, ATCC, CRL1586) were cultured in maintenance medium comprised of Dulbecco Modified Eagle Medium (DMEM; Gibco, Thermo Fisher Scientific, Waltham, Massachusetts, USA) supplemented with 10% Fetal Bovine Serum (FBS; Gibco, cat. #10270–106), penicillin (100 IU/ml), and streptomycin (100 μg/ml) (Gibco, cat. #15140–122, Life Technologies Corporation, USA) in a 5% CO_2_ environment at 37°C and passaged every 3 to 4 days in T75 tissue culture flasks (Nunc, Thermo Fisher, cat. #156499). Vero E6 cells seeded into different culture vessels for overnight incubation prior to virus infection for titration or neutralization assays were grown under the same conditions.

### Study population

The study includes 161 plasma or serum samples from PCR-confirmed SARS-CoV-2 infected adults who recovered from mild to moderate COVID-19 symptoms and did not require hospitalization. All recovered patients were infected between March and July 2020, prior to the emergence of any variants of concern in Denmark and, therefore, were infected with ancestral SARS-CoV-2 strains. Samples were collected in the same period, thus precluded exposure to subsequent infection with variants of concern or vaccination. Consent was given by all blood donors included in this study, which included use of archived samples in the validation of new methods and assay investigations as quality control projects, as is standard operations for blood centers associated with the Danish Healthcare System. Donation was carried out under national guidelines and also met the criteria of current European Union Commission Directives for donation of human blood products. Samples from 103 adults who received two doses of the mRNA vaccine BNT162b2 between January 2021 and May 2021 were collected between February 2021 and July 2021, 27 to 142 days after vaccination. The study constitutes method development for national infectious disease surveillance performed on excess biological material by Statens Serum Institut, an institute under the Danish Ministry of Health, according to Section 222 of the Danish Health Act and following data protection regulations. The study is therefore exempt from ethical review. Pre-pandemic samples collected before 2020 included 36 serum samples from different donors and, for evaluating different matrices, concurrently collected serum, EDTA plasma and sodium heparin plasma samples collected from a single donor as well as concurrently collected serum and sodium heparin plasma sample from a second donor. Samples were stored in single use aliquots and tested for SARS-CoV-2 antibodies using the SARS-CoV-2 Total antibody kit from Wantai (Beijing, China) according to the manufacturer’s instructions. Patient samples were inactivated at 56°C for one hour prior to testing in the neutralization assay and handled sterile.

### SARS-CoV-2 isolation and passage

#### Primary virus isolation

5 × 10^4^ Vero E6 cells per well were seeded in a 24-well plate and cultured overnight in maintenance medium at 37°C, 5% CO_2_. Cell culture media was removed prior to inoculation, the monolayer was washed once with 1 mL Dulbecco’s Phosphate buffered saline (DPBS; Gibco, cat. #14190–094, Thermo Fisher Scientific, Waltham, Massachusetts, USA) and covered with 250 μL infection media (DMEM and 1% Penicillin/Streptomycin). Throat swabs submerged in DPBS were used for the primary isolation of SARS-CoV-2 variants. The cells were incubated with the inoculum for 1 hour at 37°C, 5% CO_2_, followed by the addition of 1 mL maintenance medium. The cell culture supernatant was harvested 72–96 hours post-inoculation, clarified by centrifugation at 300 × g for 5 minutes, and stored at -80°C.

#### Virus passage

1.5 × 10^6^ Vero E6 cells were seeded in a 75 cm^2^ flask in maintenance medium and incubated overnight at 37°C, 5% CO_2_. The cell monolayer was washed once with 5 mL DPBS and inoculated with 25 μL Passage 1 virus diluted in 2 mL DMEM. After a 1 hour incubation with the inoculum at 37°C, 5% CO_2_, 10 mL virus growth media (DMEM, 5% FBS, 1% Penicillin/Streptomycin, and 10 mM HEPES buffer) was added and incubated at 37°C, 5% CO_2_. The virus was harvested after 72 hours by freezing the flasks at -80°C and thawing at +4°C. The passage 2 virus stock was clarified by centrifugation at 300 × g for 5 minutes and the supernatant stored in single use aliquots at -80°C.

### Virus isolates

Virus neutralization was measured for the following SARS-CoV-2 variants: early pandemic (lineage B.1) strain SARS-CoV-2/Hu/Denmark/SSI-H1 (Genbank accession number ON763516); Alpha variant (lineage B.1.1.7) strain SARS-CoV-2/Hu/Denmark/SSI-H14 (Genbank accession number ON784346); Beta variant (lineage B.1.351) strain hCoV-19/Netherlands/NoordHolland_10159/2021 (cat. #014V-04058, European Virus Archive–Global, Marseille, France); Gamma variant (lineage P.1) strain SARS-CoV-2/Hu/DK/SSI-H26 (Genbank accession number ON763756); and the Delta variant (lineage B.1.617.2) strain SARS-CoV-2/Hu/Denmark/SSI-H11 (Genbank accession number OM444216). Strains from Denmark were isolated at Statens Serum Institut from throat swabs on Vero E6 cells. All virus strains were sequenced to confirm identity, the absence of cell culture-derived mutations, and the presence of lineage-specific mutations in the spike protein.

### Virus titration

Actively growing, non-confluent Vero E6 cells within passages 5 to 20 were dissociated from a T75 tissue culture flask by removing the maintenance medium, washing the cells once with 5 mL DPBS, and overlaying the monolayer with 2 mL undiluted 10X TrypLE (Gibco, cat. #A12177-01). The dissociation media was removed immediately and the flask incubated horizontally at 37°C, 5% CO_2_ for 10 minutes. The cells were resuspended in 10 mL culture media and counted on a hemocytometer. The cell concentration was adjusted to 1 × 10^5^ cells/mL with maintenance media and 100 μL of the cell suspension transferred to each well in a 96-well plate and place in a 37°C, 5% CO_2_ incubator overnight. A ½ log_10_ (3.162-fold) serial dilution of SARS-CoV-2 viruses was prepared the following day in virus diluent that comprised DMEM culture media containing 25 mM HEPES buffer (Sigma, cat. #7365-45-9), 1% penicillin/streptomycin, 1% bovine serum albumin. The diluted virus was placed at 37°C, 5% CO_2_ for one hour to simulate conditions for the microneutralization assay. The cell monolayer prepared the preceding day was washed twice with 100 μL DPBS before adding 100 μL of diluted virus to the appropriate wells. The inoculated cells were incubated at 37°C, 5% CO_2_ for 24 hours when the titer was determined using the anti-nucleocapsid ELISA or 72/96 hours when the titer was determined by observed cytopathic effect (CPE). The 50% tissue culture infectious dose (TCID50) was calculated with the Reed and Muench method [19].

### Microneutralization

Vero E6 cells were seeded in a 96-well tissue culture plate as described for the virus titration. The following day, a two-fold serial dilution of heat-inactivate serum/plasma samples were prepared in virus diluent (formulation described in section 2.5). 60 μL diluted serum was mixed with 60 μL virus diluent containing 300 × TCID_50_ SARS-CoV-2 virus (as titered after 96 hours). The solution was incubated for 1 hour at 37°C, 5% CO_2_ to allow opsonization. The cell monolayer was washed twice with 100 μL DPBS, and 100 μL serum-virus mixture added gently to the wells. The inoculated cells were incubated at 37°C, 5% CO_2_ for 24 hours. Quadruplicate wells containing cells with 300 × TCID_50_ SARS-CoV-2 virus without serum (virus control) in 100 μL and quadruplicate wells containing cells with 100 μL virus diluent only (cell control) were included on each microneutralization plate.

The 50% virus infection cutoff was calculated from the virus control wells and the cell control wells as follows:

$$50\%\;virus\;infection\;cutoff=\frac{\left(average\;OD\;of\;virus\;control\;wells)+(average\;OD\;of\;cell\;control\;wells\right)}{2}$$

All values below or equal to the cutoff value were considered positive for neutralization activity. The reciprocal serum dilution corresponding to the highest serum dilution positive for neutralization activity is reported as the categorical 50% neutralization antibody titer. Exact 50% neutralization antibody titers were calculated as the intercept of the 50% cutoff value with a four-parameter logistic regression curve fitting over the eight point serum/plasma titration. Assay repeatability and intermediate precision were calculated for three plasma pools with either a low neutralization titer, medium neutralization titer, or high neutralization titer using Data Sheet S3 provided by Andreasson et al. 2015 [20].

### ELISA

#### Antibodies

The following antibody combinations were evaluated in the SARS-CoV-2 infected cell culture ELISA: rabbit anti-SARS-CoV-2 nucleocapsid protein monoclonal antibody (mAb) (1:2500 dilution; cat. #40143-R019, Sino Biological, Beijing, China) with secondary antibody mouse anti-rabbit IgG (γ-chain specific) peroxidase-conjugated monoclonal antibody clone RG-96 (1:6000 dilution; cat. #A1949, Sigma Aldrich, Merck, Darmstadt, Germany); or mouse anti-SARS-CoV-2 nucleocapsid protein mAb clone 7E1B (1:4000 dilution; cat. #BSM-41414M, Bioss Inc, Woburn, MA, USA) with secondary antibody goat anti-mouse IgG peroxidase-conjugated polyclonal antibody (1:10000 dilution, cat. #A16078, Invitrogen, Thermo Fisher). The optimal antibody dilutions were determined empirically (S1 Fig). The rabbit mAb was used in experiments performed at the start of the pandemic when reagents for SARS-CoV-2 were limited. Once available, the mouse mAb was used as the preferred detection antibody due to a better signal-to-background ratio (S1 Fig).

#### Anti-nucleocapsid ELISA assay

The culture media was removed from the 96-well tissue culture plate wells with inoculated and mock infected Vero E6 cell monolayers. The cells were washed twice with 100 μL DPBS, taking care to not dislodge the cells. The SARS-CoV-2 infected monolayer was fixed and permeabilized with cold 80% (v/v) acetone in DPBS for 10 minutes to expose the internal nucleocapsid protein. Incubation with fixative for up to 25 minutes did not affect antigen availability (S2 Fig). The fixative was subsequently removed and the wells air dried. The plates were washed three times with wash buffer (1% (v/v) Triton-X100, 0.2% Tween20 in DPBS) for 30 seconds and incubated with 100 μL primary antibody for five minutes on an orbital shaker (300 rpm) at room temperature and subsequently for one hour at 37°C. The plates were washed as before and incubated with the secondary antibody for five minutes on an orbital shaker (300 rpm) at room temperature and subsequently for one hour at 37°C. The plates were washed five times with wash buffer for 30 seconds, followed by three washes with deionized water without soaking. 100 μL TMB ONE substrate (Kem-en-tec, cat. #4380) equilibrated to room temperature was added to each well and incubated for 10 minutes at room temperature in the dark. The reaction was stopped by the addition of 100 μL 0.2 M H_2_SO_4_ and the absorbance read at 450 nm with 620 nm as reference on a FluoStar Omega plate reader (BMG Labtech, Offenburg, Germany). Resume by washing the plates three times with wash buffer before adding the primary antibody for the ELISA. In the present study, the ELISA was done directly after fixing and air drying the tissue culture plates.

### Normalization and calculations

To reduce inter-assay variation, titers were normalized using two different models:

Model 1

$$x\times ((\frac{x-1}{Log(1280)-1}\times \frac{Pos_{nom}-Pos}{Pos})+1)$$

where *x* is the log_10_ transformed titer of the sample. *Pos* is the log_10_ titer of the positive control included on the same assay plate as the sample. *Pos*_*nom*_ is the average log_10_ titer of the positive control determined from eight optimal runs denoted as the nominal value of the positive control material. The second fraction calculates the bias of the result from the positive control according to the nominal “expected” value. Thus, if the positive control in a given run has a titer identical to the nominal value, samples will not be corrected. The first fraction describes the relative size of the titer of the sample compared to the maximum titer in a normal assay (here 1:1280). This weighs each titer according to size. Range is adjusted according to the minimum positive value (log_10_ 10 = 1) to exclude normalization for positive samples at the minimum titer of 10, which is the lower limit of quantification. This exclusion is designed to diminish influence of normalization on the qualitative judgement of borderline samples. The correction increases with titer and is greatest at maximal titer (1280). Model 1 assumes that variation and titer size has a linear relation. Effect of the normalization evolves linearly from 0 correction at titer 1:10 to maximal correction in an assay with a maximum titer of 1:1280.

Model 2

$$x\times (((0.3038x^{2}-0.7468x+0.4431)\times \frac{Pos_{nom}-Pos}{Pos})+1)$$

This model normalizes virus neutralization titers using a polynomial titer-weighing fraction as calculated from the precision study on low, medium, and high titered samples. It is dependent on precision data from each performing laboratory and should be determined empirically. The precision study showed that when the positive control is affected by a suboptimal assay, the correspondent effect on the samples can be described in this 2^nd^-degree polynomial curve. Substituting the linear titer-weighing fraction from model 1 with this polynomial equation gives model 2. In model 2, *x* is log (log_10_) of the titer of the sample. *Pos* is the log_10_ titer of the positive control included on the same plate. *Pos*_*nom*_ is the nominal value of the positive control. It is designed to exclude correction at titer 10 (polynomial curve is forced through 0 at titer 10), rendering it less effective in the low end, however with a resultant intentionally diminished influence on the qualitative judgement in the assay. Omitting this strategy, fitting the model solely on the precision study data, improves precision in the low end; however, with the potential of creating an undesired mismatch in qualitative judgement between non-normalized data and normalized data.

### Statistical analysis

The total area under the curve was calculated using the mean optical density (OD) values of replicate measurements plotted relative to the log_10_ decimal virus dilution. Comparison between neutralization titers for virus variants was performed using the Wilcoxon rank sum test. Statistical analyses and graphing were done with GraphPad PRISM version 8.3.0. (GraphPad Software Inc., San Diego, CA).

## Results

### Optimal duration of virus incubation

**Fig 1:**
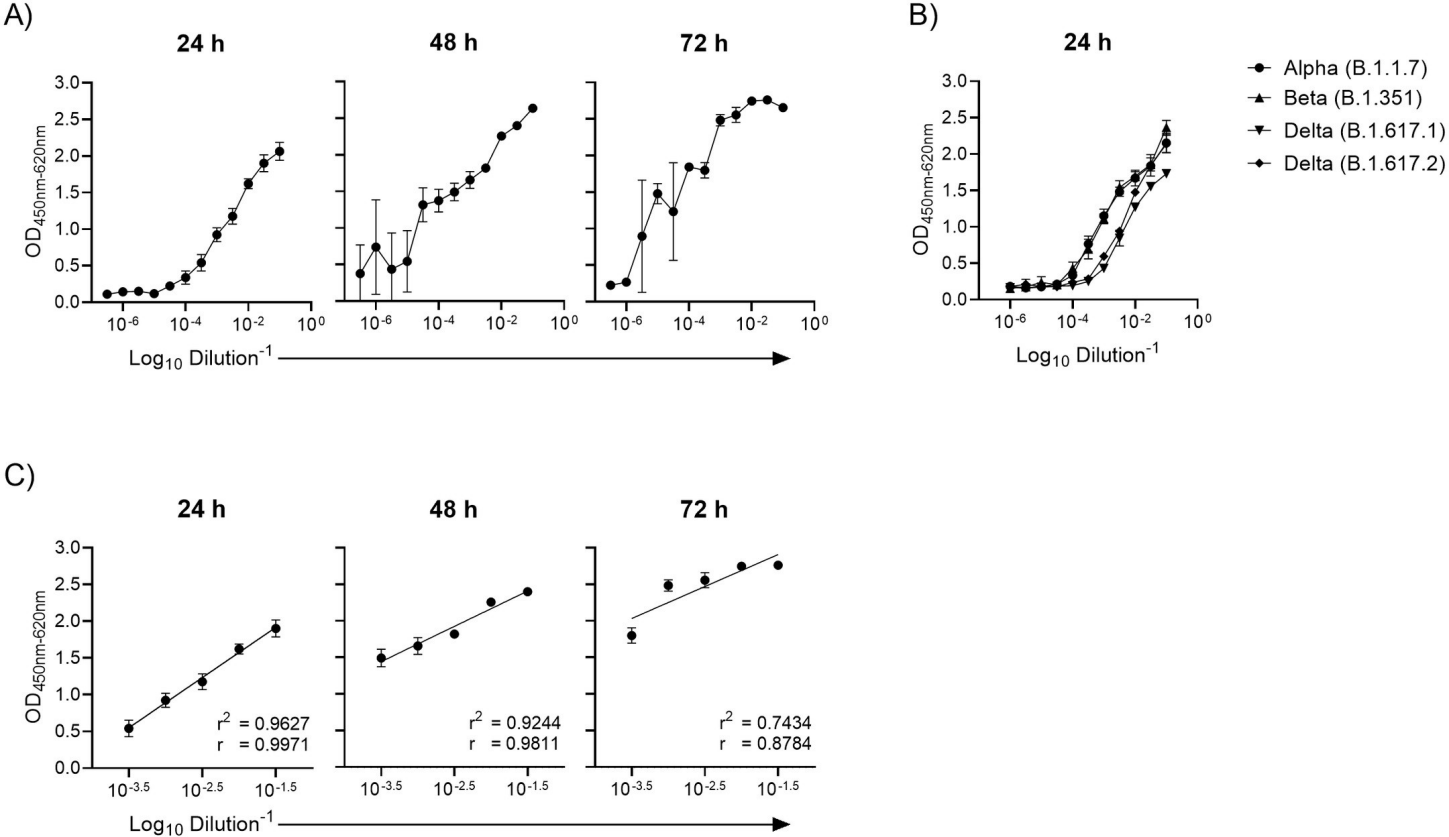
Relationship between virus inoculum size and SARS-CoV-2 nucleocapsid protein measured at 24, 48 and 72 hours post-inoculation. (A) A serial dilution of SARS-CoV-2 early pandemic strain (lineage B.1) was transferred from a master stock to three identical cell culture plates seeded with 10^4^ Vero E6 cells per well the preceding day. An anti-SARS-CoV-2 nucleocapsid ELISA was performed on washed and fixed cells after 24, 48 and 72 hours. Each data point represents the mean of quadruplicate measurements with the standard deviation. ELISA primary antibody: rabbit mAb 40143-R019. (B) Different SARS-CoV-2 variants of concern treated as described in (A) show a similar linear relationship between inoculum and nucleocapsid protein levels measured by ELISA 24 hours post-inoculation. The comparison between strains was done on the same day using a single dilution for each serum sample. ELISA primary antibody: mouse mAb BSM-41414M. (C) Linear regression and Pearson correlation test of virus inoculum size and SARS-CoV-2 nucleocapsid protein level in the quantification range from a dilution of 10^−3.5^ to 10^−1.5^. r^2^ = Goodness of fit. r = Pearson correlation coefficient. Error bars represent standard deviations of replicate determinations.

To determine the optimal incubation period between virus inoculation and quantitation of the SARS-CoV-2 nucleocapsid protein by ELISA, a single dilution series of a Danish clinical isolate was titrated on replicate plates and the level of nucleocapsid protein measured at 24h, 48h and 72h post-inoculation. The level of nucleocapsid protein directly correlated with the inoculum with a maximum correlation after 24 hours (Pearson r = 0.9971) decreasing to 0.9811 and 0.8784 after 48 hours and 72 hours, respectively (Fig 1C). The linearity between inoculum and nucleocapsid ELISA OD values, measured as a goodness of fit in a linear regression curve fit of the quantification range of the dilution series, was highest at 24 hours (r^2^ = 0.9627) compared to 48 hours (r^2^ = 0.9244) and 72 hours (r^2^ = 0.7434). Overall, lower variance between quadruplicate measurements also occurred at 24 hours post-infection. The correlation between virus inoculum size and nucleocapsid ELISA OD values was similar for three SARS-CoV-2 variants of concern that include Alpha (B.1.1.7; r = 0.9830), Beta (B.1.351; r = 0.9722), and Delta (B.1.617.1; r = 0.9927, and B.1.617.2; r = 0.9954) (Fig 1B). With optimal linearity and the lowest variance, the 24 hour incubation period was selected as the standard viral infection incubation time for the SARS-CoV-2 virus neutralization assay.

### Optimal time point for determining virus titers

Four distinct early pandemic clinical isolates were titrated on Vero E6 monolayers, seeded as 10000 cells per well the day prior to inoculation, and the virus titers determined at 24 hours with the anti-nucleocapsid ELISA (CPE is indiscernible at this time) and at 72 and 96 hours with CPE read-out. To confirm an equal sensitivity for CPE and ELISA read-out at later time points, an ELISA was performed at 96 hours after CPE observation and were 100% concordant with CPE for identifying virus positive wells. The TCID_50_/mL titers of three out of four isolates plateaued at 72 hours post-inoculation, while the titer of one isolate continued to increase up to 96 hours post-inoculation (S3 Fig). The proportional increase from 24 hours measured by ELISA to 96 hours by CPE varied from 17 to 363-fold (S3 Fig). Taken together, these observations suggest that different growth kinetics affect virus titers measured at different time points. We therefore selected a virus titration incubation period of 96 hours to allow sufficient time for slower growing isolates to replicate.

### Optimal Vero E6 cell number and morphology for virus infection

We next investigated different cell-specific conditions for viral infection that include cell number and infection in suspension or attached to the cell culture plate in a monolayer. For the latter, Vero E6 cells were seeded as 6000, 8000, 10000, 12000, 14000, or 16000 cells per well in a 96-well tissue culture plate 24 hours before inoculation with SARS-CoV-2. On the day of infection, the cell numbers increased to 12000, 14000, 16000, 18000, 20000, and 22000, respectively, as determined from dissociated cells from quadruplicate wells. For the suspension infection, the corresponding number of cells in the monolayers on the day of infection were used. All other variables were kept constant for both the monolayer and suspension infections i.e. cell passage, virus stock, and prepared reagent stocks.

**Fig 2:**
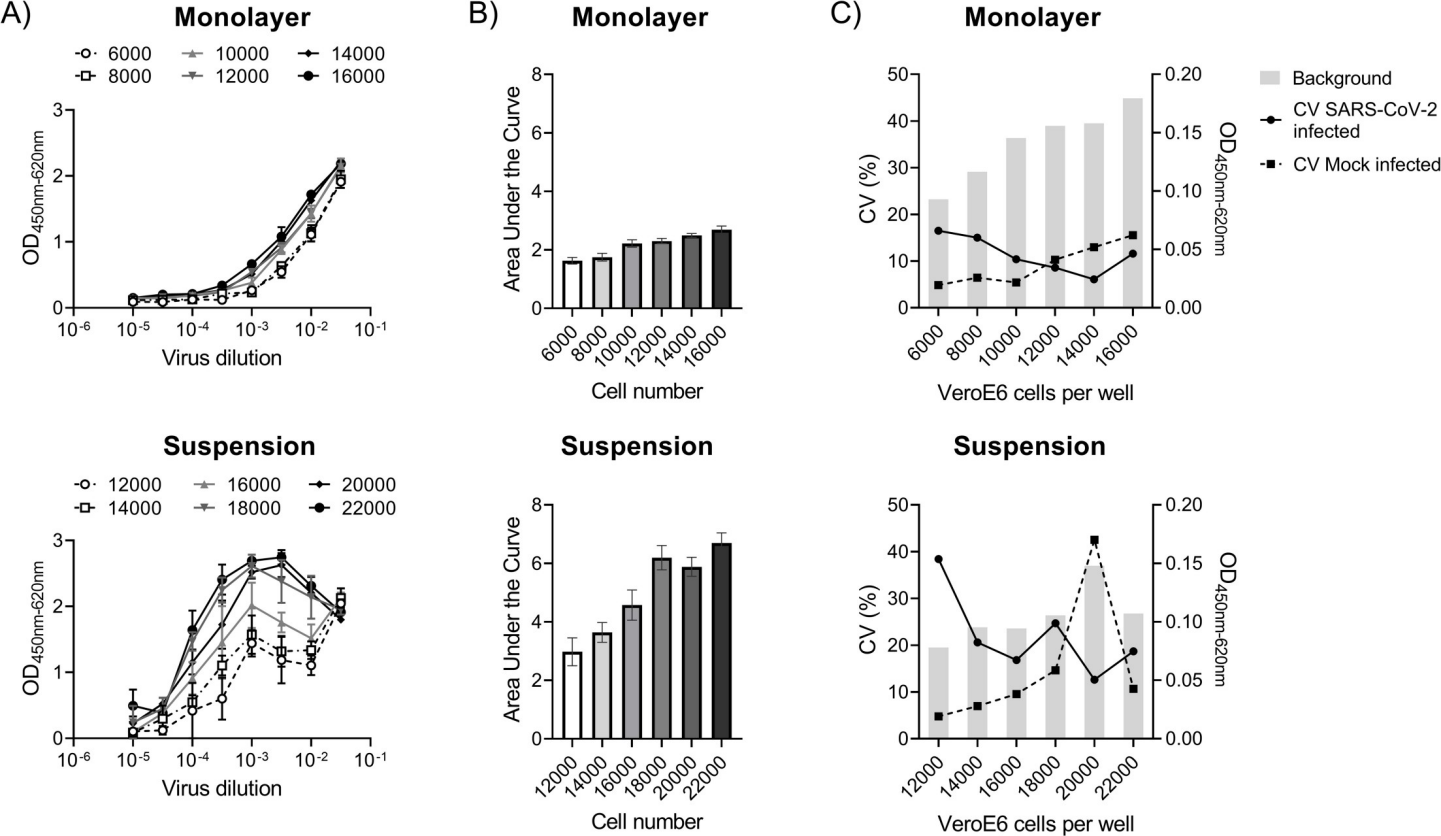
Effect of Vero E6 cell number and morphology on the relationship between SARS-CoV-2 nucleocapsid protein measured by ELISA and SARS-CoV-2 virus inoculum. Triplicate serial dilutions (0.5 log_10_-fold) of a Danish SARS-CoV-2 clinical isolate (early pandemic strain (lineage B.1)) was added to increasing numbers of Vero E6 cells in a monolayer or in suspension. The cell numbers indicated for the monolayer are as seeded the day prior to the infection. On the day of infection, the seeded cell numbers– 6000, 8000, 10000, 12000, 14000, and 16000 –increased to 12000, 14000, 16000, 18000, 20000, and 22000, respectively. The latter cell numbers were used for the infection of suspended cells to ensure a similar multiplicity of infection for the monolayers. (A) SARS-CoV-2 virus titration curve for different cell numbers infected in a monolayer and suspension. Symbols indicate the mean and error bars the standard deviation. (B) The overall SARS-CoV-2 nucleocapsid protein signal measured for the virus dilution series, expressed as area under the curve for the Log_10_ decimal virus dilution and ELISA OD value as a measure of the magnitude of variance associated with changes in cell number (shown in A). Error bars represent the 95% confidence interval. (C) The percentage coefficient of variation (CV) for SARS-CoV-2 infected and mock infected cells as well as the background anti-SARS-CoV-2 nucleocapsid protein level measured for mock infected cells. For all experiments, ELISA primary antibody: rabbit mAb 40143-R019.

Compared to cells infected in suspension, the infection of a monolayer resulted in a more confined linear relationship between the SARS-CoV-2 nucleocapsid protein level measured in ELISA and the inoculum (Fig 2A). Overall, higher Vero E6 cell numbers associated with increased anti-nucleocapsid ELISA signal measured as area under the virus dilution curve (AUC), with a greater effect for cells infected in suspension (Fig 2B). For monolayers, the AUC had a modest increase from 1.63 to 2.69 for 6000 to 16000 cells seeded per well (corresponding to 12000 to 22000 cells on the day of infection). In contrast, the AUC for cells infected in suspension increased from 2.98 to 6.69 for 12000 to 22000 cells infected. Compared to cells infected in suspension, the monolayer infections had an overall lower signal variability among replicates with coefficients of variants less than 20% for infected and mock infected cells at all cell numbers tested (Fig 2C). From these observations, infection of a monolayer was deemed more robust and selected for further assay optimization.

### Optimal Vero E6 cell number for virus neutralization assay sensitivity

**Fig 3:**
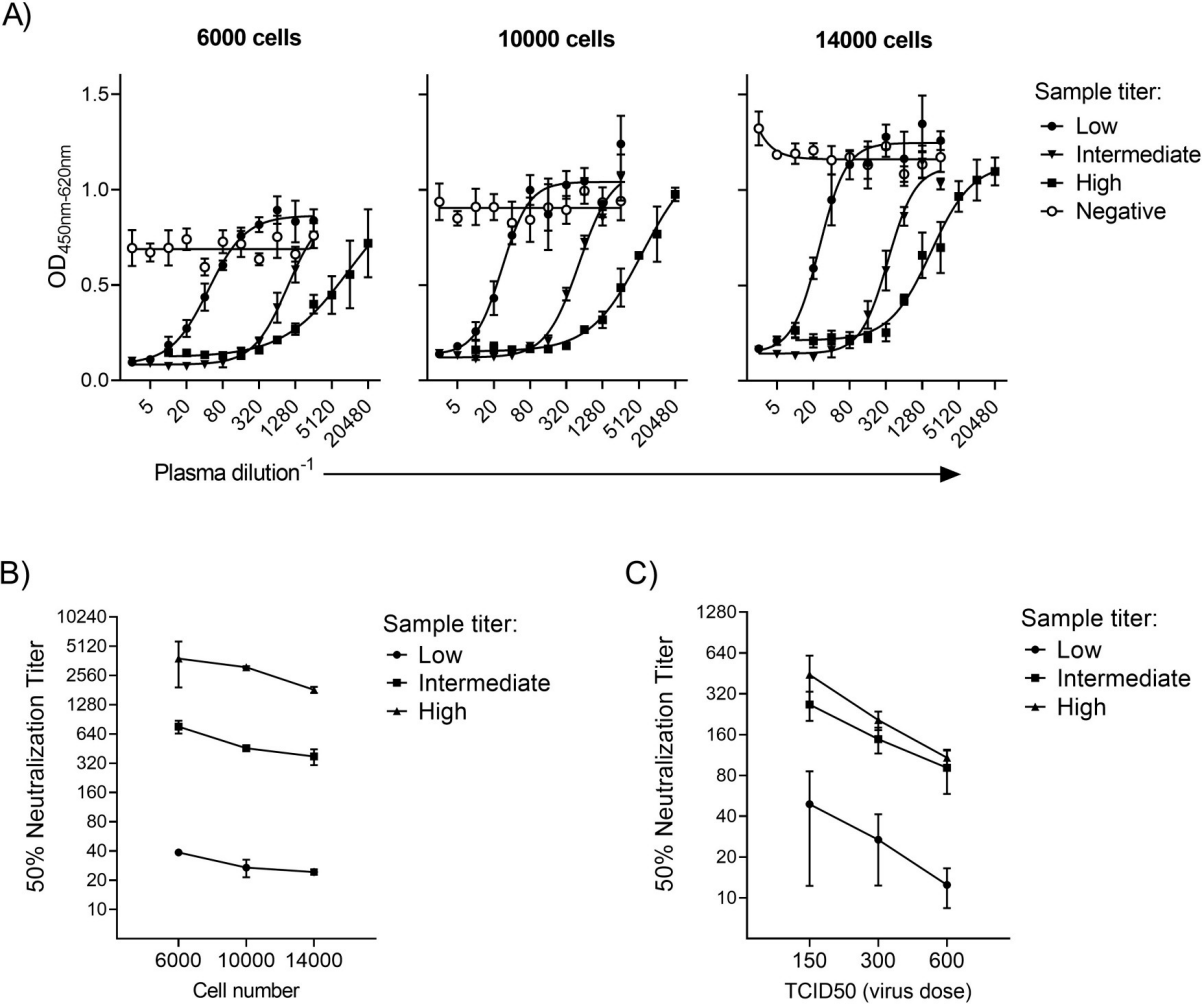
Effect of variable Vero E6 cell number and virus amount on assay sensitivity. (A) Four serum samples of known neutralization titer (negative, 1:10, 1:160 and 1:1280) were tested in the neutralization assay (early pandemic strain (lineage B.1)) with different Vero E6 cell numbers seeded the day prior (6000, 10000, or 14000). Lines represent the four-parameter titration curve calculated from the mean of triplicate measurements. (B) Neutralization titers for assays using 6000, 10000 and 14000 cells seeded the day prior. (C) Neutralization titers for three samples from assays using 10000 cells and different amounts of input virus (150, 300 and 600 TCID_50_). Each data point represent the mean of triplicate determinations with standard deviation. For all experiments, ELISA primary antibody: rabbit mAb 40143-R019.

The effect of variable Vero E6 cell number on the neutralization assay was assessed by seeding 6000, 10000, or 14000 cells/well into 96 well tissue-culture plates the day before the assay. Four serum samples with negative, low, medium, or high SARS-CoV-2 neutralization titers, were tested in triplicate for each cell number. As expected, the signal for the negative serum and the upper plateau of all the positive sera increased with increasing cell numbers (Fig 3A). For the low titered sample, titers were 38, 26, and 24 for 6000, 10000 and 14000 cells/well; for the medium titered sample, titers were 759, 456 and 346 for 6000, 10000 and 14000 cells/well; and for the high titered sample, titers were 3176, 3086 and 1755 for 6000, 10000, and 14000 cells/well, respectively (Fig 3B). A cell density of 10000 cells/well was chosen as standard for the assay based on a low variation and a moderate cell consumption.

These studies provide insight into the impact an unintentionally increase/ decrease of up to 40% from a target cell density of 10000 cells. Variations in cell number ± 40% did not affect the negative sample which was consistently tested negative. The positive samples were affected by a 40% deviation, however the titer was not altered by more than a maximum of one titer step in the entire range. Thus, a variation in cell density to a degree that can be expected in routine analyses, is not expected to have a significant influence on assay performance.

### Effect of virus concentration on assay sensitivity

Serum samples with low, medium and high neutralization titers were tested in triplicate with 150 TCID_50_, 300 TCID_50_, and 600 TCID_50_ virus as titered at 96 hours. For each serum sample, titers decreased with increasing virus dose (Fig 3C). Reducing the virus dose from 300 TCID_50_ to 150 TCID_50_ resulted in a two-fold increase in titer equally in the low, medium and the high titered sample. Inversely, increasing the dose from 300 TCID_50_ to 600 TCID_50_ resulted in halving of titers in all three sample categories. Variations in virus concentration to the extent that can occur in an everyday run, is therefore unlikely to have any effect exceeding normal analytical variation.

### Optimization of results—normalization

Our precision analysis indicated an association between variation and titer. We observed that factors influencing variation of the positive control had a similar influence on the samples results proportional to the titer size. This heteroscedastic behavior could be accounted for by taking the calculated titer size into consideration with the calculated bias of the positive control. Therefore, we developed two methods to normalize neutralization titers relative to the same positive control included all assays. The normalization methods are both weighted according to titer in order to increase correction with increasing titers measured. The effect of result optimization through the use of the normalization models are described under Assay evaluation.

### Assay recommendations

Based on our experience, we formulated the following recommendations for system suitability tests (SSTs): i) mean of the OD values from the virus controls must be equal to or above 1.0; ii) signal to noise ratios calculated as the virus controls divided by the cell controls are preferably above 6; iii) the exact titer of the positive control material should not exceed 1.5 times the nominal value determined from at least five optimal assays. These criteria may differ across laboratories; thus, SSTs should be evaluated by each performing laboratory in order to apply appropriate acceptance criteria for the continuous monitoring of assay performance over time.

### Assay performance characterization

#### Precision

To evaluate assay precision as described [20], we tested SARS-CoV-2 convalescent plasma pools with either low, medium or high neutralization titers in the neutralization assay as five replicates each on five different days. The mean titer for the low titer sample was 54 (range: 42–85; median categorical titer 40, range: 20–80), for the medium titer sample 199 (range: 135–316; median categorical titer 160, range: 160–320), and the high titer sample 1109 (range: 510–2310; median categorical titer 640, range: 640–2560). Repeatability within assays was 12.6%, 14.0%, and 22.4% for the low, medium and high level pools, respectively. Intermediate precision between assays was 20.9%, 26.5% and 63.1% for the low, medium and high level pools, respectively.

**Fig 4:**
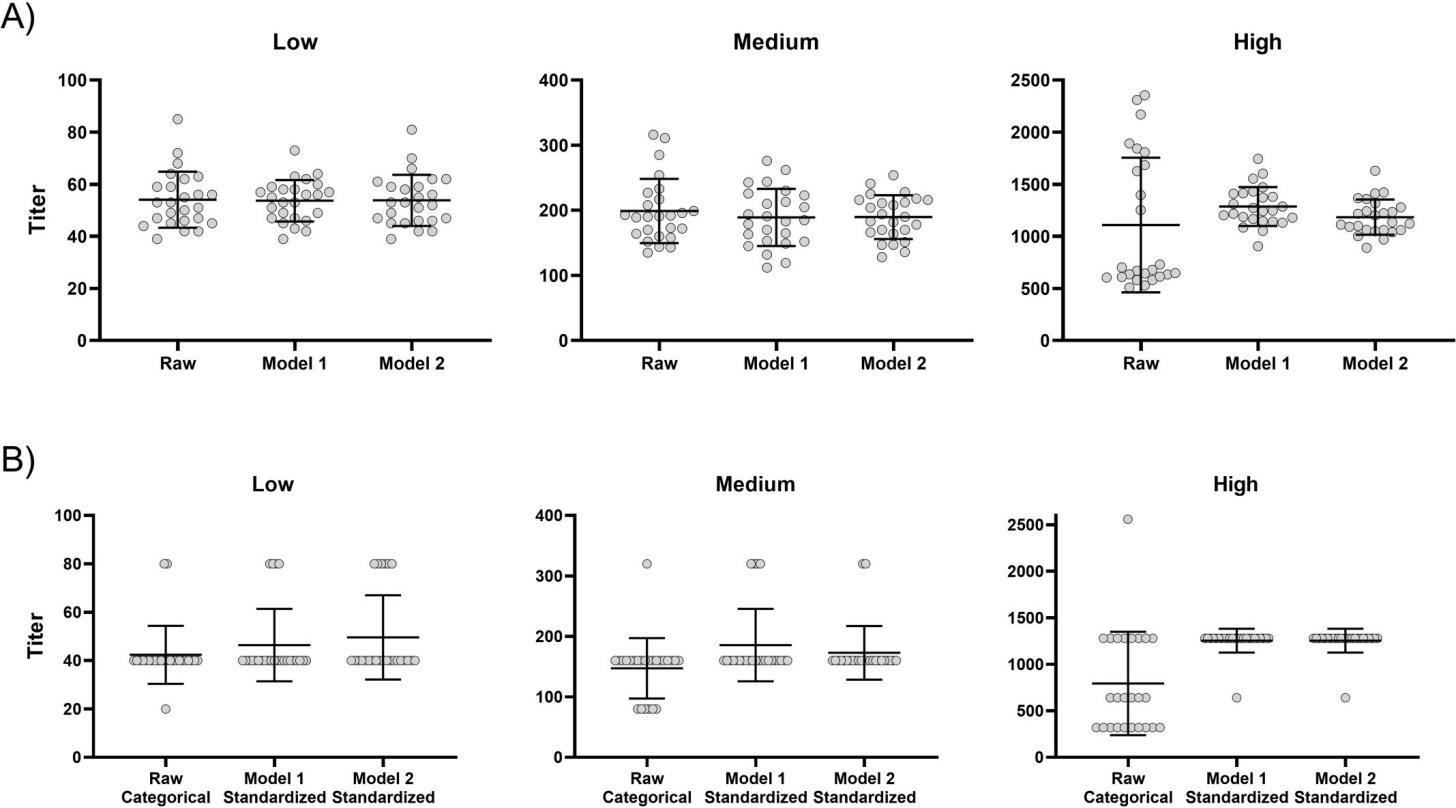
Distribution of raw and normalized results from a precision study. Scatterplots of exact titers (A) and categorical/ standardized titers (B) from replicate determinations in the low, medium and high titered sample groups from raw data, data normalized using model 1 and data normalized using model 2. Bars represent global mean and standard deviation. Exact titer = titer calculated according to a four-parameter logistic regression curve fit from non-normalized (raw data) or normalized OD-values; Categorical titer = the highest serum dilution positive for neutralization activity determined from non-normalized OD-values (raw data); Standardized titer = the closest standard dilution factor on a log_2_ scale starting from a 1:10 dilution calculated from normalized OD-values. Virus: early pandemic strain (lineage B.1). For all precision study experiments, ELISA primary antibody: rabbit mAb 40143-R019.

Normalization of data using model 1 changed repeatability from 12.6%, 14.0% and 22.4% to 12.3%, 14.4% and 14.3% in the low, medium and high titered samples, respectively. Intermediate precision is reduced from 20.9%, 26.5% and 63.1% in the low, medium and high titered samples to 15.2%, 24.7% and 14.6%, respectively. Results from raw data and normalized data are summarized in Table 1 and individual titers listed in S1 Table. Applying the aforementioned SST recommendations on data normalized through the use of model 1, can further reduce intermediate precision in the low and the medium titered range to 13.9% and 17.8%, respectively. Normalization of data using model 2 reduced repeatability from 12.6%, 14.0% and 22.4% in the low, medium and high titered samples to 12.4%, 13.8% and 13.7%, respectively. Intermediate precision is reduced from 20.9%, 26.5% and 63.1% in the low, medium and high titered samples to 19.2%, 18.4% and 14.4%, respectively. Applying the SST recommendations on data normalized through the use of model 2, can further reduce intermediate precision in the low and the medium titered range to 13.1% and 16.5% respectively. Neither model significantly alter the pool mean titer value over five assays with five replicates in each assay (Fig 4). Normalization condenses data both intra-serially and inter-serially improving standardization and unification of results.

**Table 1:** Calculations of precision on raw data and on normalized data.

			Low	Medium	High
**Raw data**	Pool	Mean	54	199	1109
	Repeatability	s_r_	**6.8** (6.2)	**27.8** (25.5)	**248.5**
		%CV_r_	**12.6** (12.5)	**14.0** (14.1)	**22.4**
	Intermediate precision	s_Rw_	**11.3** (6.6)	**52.7** (26.7)	**699.7**
		%CV_Rw_	**20.9** (13.1)	**26.5** (14.8)	**63.1**
**Normalized–Model 1**	Pool	Mean	54	189	1286
	Repeatability	s_r_	**6.6** (6.5)	**27.3** (32.4)	**184.1**
		%CV_r_	**12.3** (12.6)	**14.4** (15.3)	**14.3**
	Intermediate precision	s_Rw_	**8.2** (7.2)	**46.7** (37.8)	**187.9**
		%CV_Rw_	**15.2** (13.9)	**24.7** (17.8)	**14.6**
**Normalized–Model 2**	Pool	Mean	54	190	1184
	Repeatability	s_r_	**6.7** (6.3)	**26.2** (30.0)	**162.7**
		%CV_r_	**12.4** (12.5)	**13.8** (15.3)	**13.7**
	Intermediate precision	s_Rw_	**10.3** (6.6)	**35.0** (32.4)	**169.9**
		%CV_Rw_	**19.2** (13.1)	**18.4** (16.5)	**14.4**

Repeatability and intermediate precision calculated for the low, medium, and high titer plasma pool by the use of Data Sheet S3 according to Andreasson et al 2015 [20]. Data in brackets are results obtained from datasets filtered according to the SST recommendations set in this article. All tests performed on the high titered samples complied with the SST recommendations.

#### Evaluation of assay specificity

**Fig 5:**
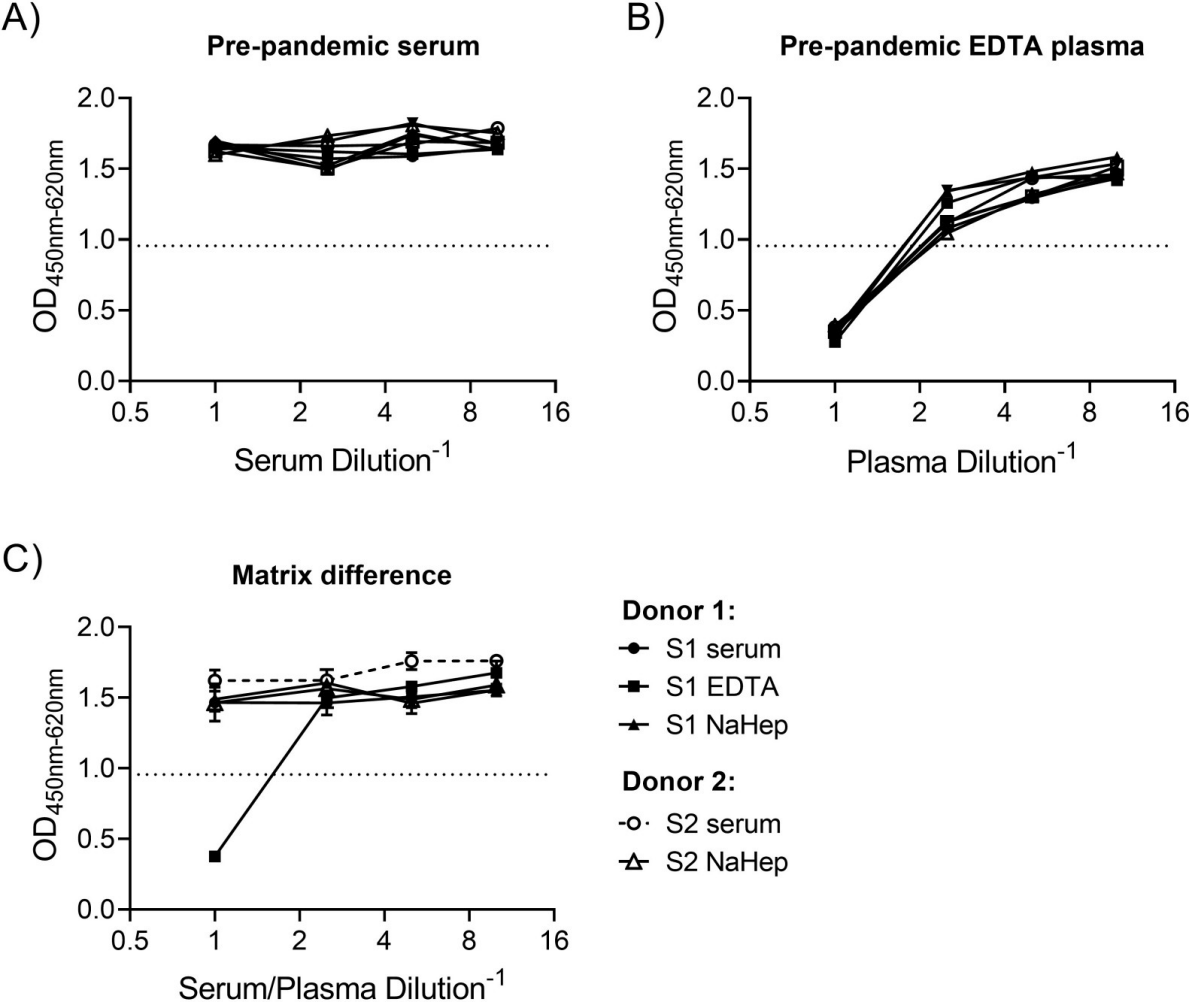
Sample matrix influence on specificity. (A) Neutralization activity on 8 negative pre-pandemic serum samples, and (B) 8 negative EDTA plasma samples run in duplicates and run undiluted, 1:2.5, 1:5 and 1:10. (C) Neutralization activity on serum, EDTA or sodium heparin (NaHep) samples collected from two donors on the same day tested in duplicate and run undiluted, 1:2.5, 1:5 and 1:10. Virus: early pandemic strain (lineage B.1). Error bars represent standard deviations of duplicate determinations. ELISA primary antibody: rabbit mAb 40143-R019.

To evaluate assay specificity, we identified the lowest serum/plasma dilution at which non-specific factors that result in false positive reactions or antibody-independent virus neutralization were diluted out. We selected pre-pandemic serum samples (n = 8) and EDTA plasma samples (n = 8) to be tested undiluted, and diluted 1:2.5, 1:5, and 1:10 (Fig 5A and 5B). No non-specific virus neutralization effects at low dilutions or undiluted was observed in the SARS-CoV-2 antibody negative serum samples. In contrast, a clear non-specific virus inhibition, with a mean reduction of 79.5%, was observed in the SARS-CoV-2 antibody negative EDTA plasma samples when tested undiluted. This inhibition was less pronounced at a plasma dilution of 1:2.5 (30.3%) and 1:5 (18.6%) and near completely titrated out at 1:10 (12.4%). Conducting a similar test with a serum sample, an EDTA plasma sample, and a sodium heparin plasma sample taken from the same donor at the same time point, the EDTA plasma sample was the only sample matrix displaying a false positive result when tested undiluted (Fig 5C). Recommended pre-dilution for optimal quantification is therefore 1:10 for EDTA plasma samples and can be as low as 1:1 for serum and sodium heparin.

#### Inter-operator variability

**Fig 6:**
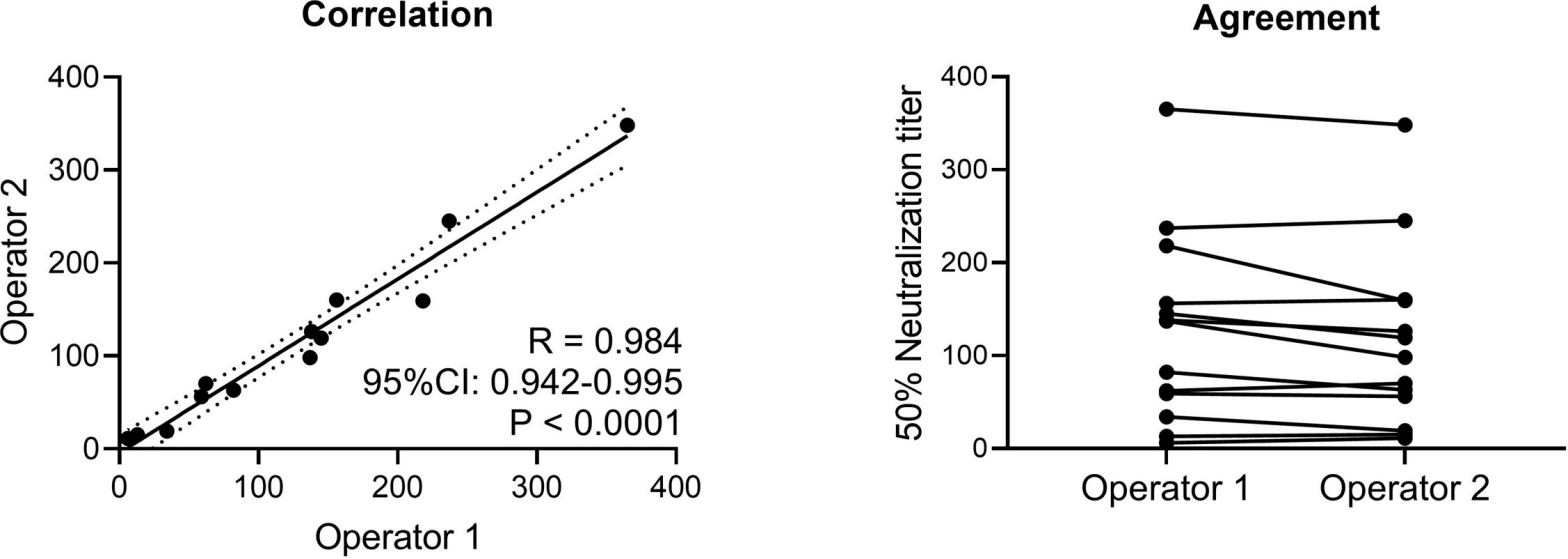
Operator-derived variation. Two operators tested the same clinical samples. Correlation and agreement between neutralization titers measured were assessed. ELISA primary antibody: rabbit mAb 40143-R019.

The precision study was performed by a single operator, hence repeatability and intermediate precision does not include technician derived variation. To test the assays robustness with regards to technician derived variation, thirteen positive samples were tested in parallel by two different operators (Fig 6). The two different operators performed the assay on the same day, using the same reagent lot numbers varying solely technician operations. Neutralization titers from both operators showed a significant high correlation (Spearman R = 0.984; 95% confidence interval: 0.952–0.995; p < 0.0001) and good agreement.

### Qualitative and quantitative assay evaluation

**Fig 7:**
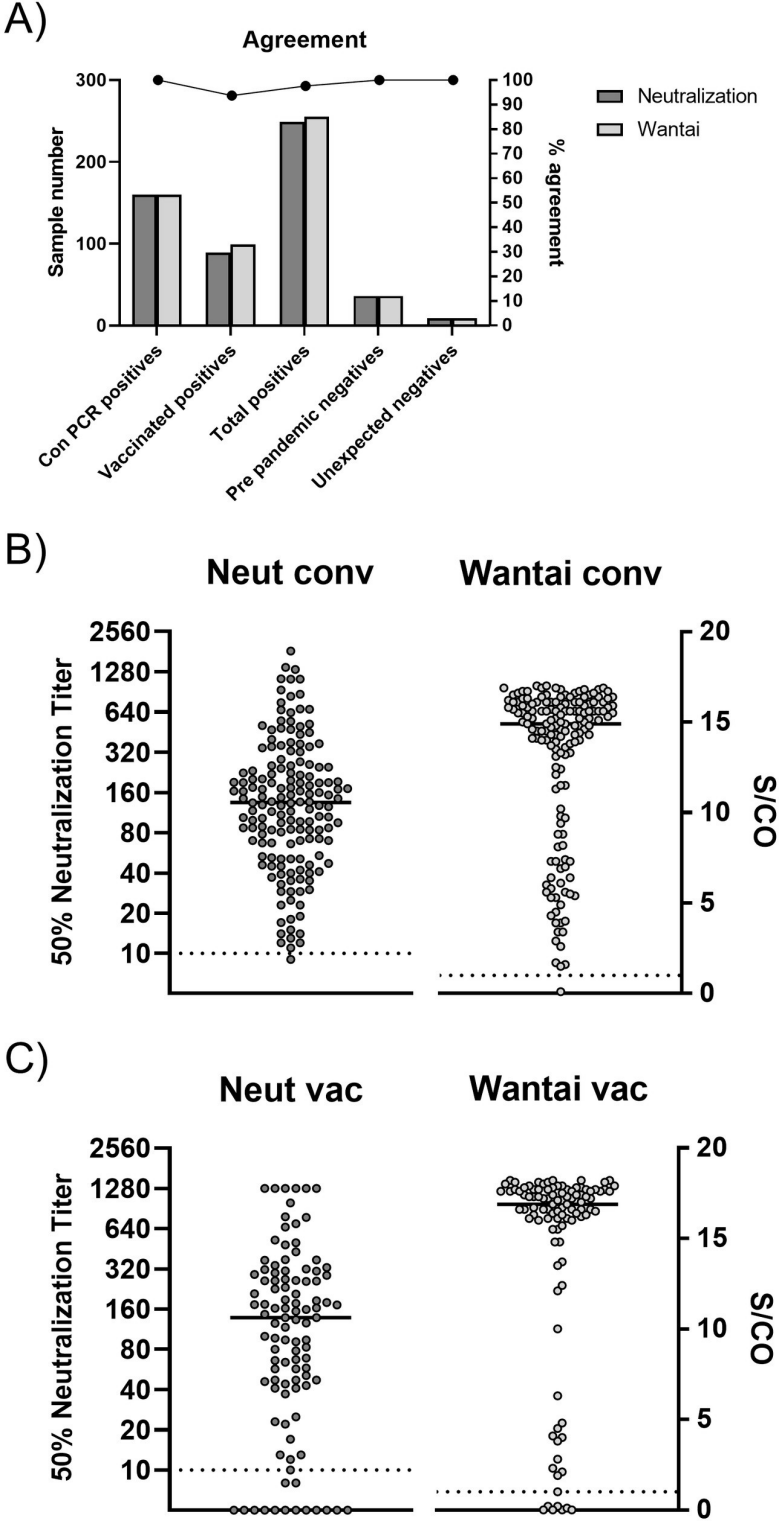
Agreement and data distribution of neutralizing antibody and total antibody results. Qualitative result comparison between SARS-CoV-2 virus neutralization qualitative results and Wantai Total Antibody ELISA kit results for the cohort of PCR confirmed positive convalescent plasma samples, SARS-CoV-2 vaccinated individuals and pre-pandemic samples (A). Distribution of 50% neutralization titers from the neutralization assay and sample over cut off (S/CO) from the Wantai ELISA from convalescent plasma (B) and SARS-CoV-2 vaccinated individuals (C). Bars represent mean, dotted lines represent assay cut off. ELISA primary antibody: (B) rabbit mAb 40143-R019; (C) mouse mAb BSM-41414M.

We evaluated the assay’s ability to correctly identify SARS-CoV-2 antibody positive and negative samples on a panel of 161 convalescent plasma and serum samples from SARS-CoV-2 PCR-confirmed patients with mild to moderate symptoms, 103 SARS-CoV-2 vaccinated individuals who received two doses of the mRNA vaccine BNT162b2 (Pfizer/BioNTech), and 36 negative serum/plasma samples collected from donors before the pandemic using the D614G ancestral strain. Seropositivity for anti-SARS-CoV-2 spike antibodies was determined using the Wantai Total Antibody ELISA kit, a highly sensitive [21] non-functional antibody binding assay that detects total antibody (IgG, IgM, IgA, IgE, IgD) against the receptor binding domain of SARS-CoV-2 spike. Results are shown in Fig 7. Of the 161 samples from SARS-CoV-2 patients who recovered from infection with ancestral strains, 160 (99.4%) tested positive for both SARS-CoV-2-specific binding antibodies in the Wantai Total Antibody ELISA assay and for virus neutralizing antibodies. The 50% virus neutralization titers ranged from 9 to >1280 (median: 135; interquartile range: 63–245). Of the 103 vaccine sera, 95 (92.2%) tested positive for RBD antibodies in the Wantai Total Antibody ELISA kit, of which 89/95 (93.7%) tested positive for neutralizing antibodies. The 50% virus neutralization titers ranged from 10 to >1280 (median: 163; interquartile range: 65–313). The one convalescent sample and eight vaccine samples that tested negative in the ELISA were also negative for neutralizing antibodies. Collectively, 249 out of 255 (97.6%) convalescent plasma and vaccine serum samples that tested SARS-CoV-2 antibody positive in the Wantai Total Antibody ELISA assay were positive for SARS-CoV-2 neutralizing antibodies. All 36 pre-pandemic samples tested negative for neutralizing antibodies.

### SARS-CoV-2 virus variants

**Fig 8:**
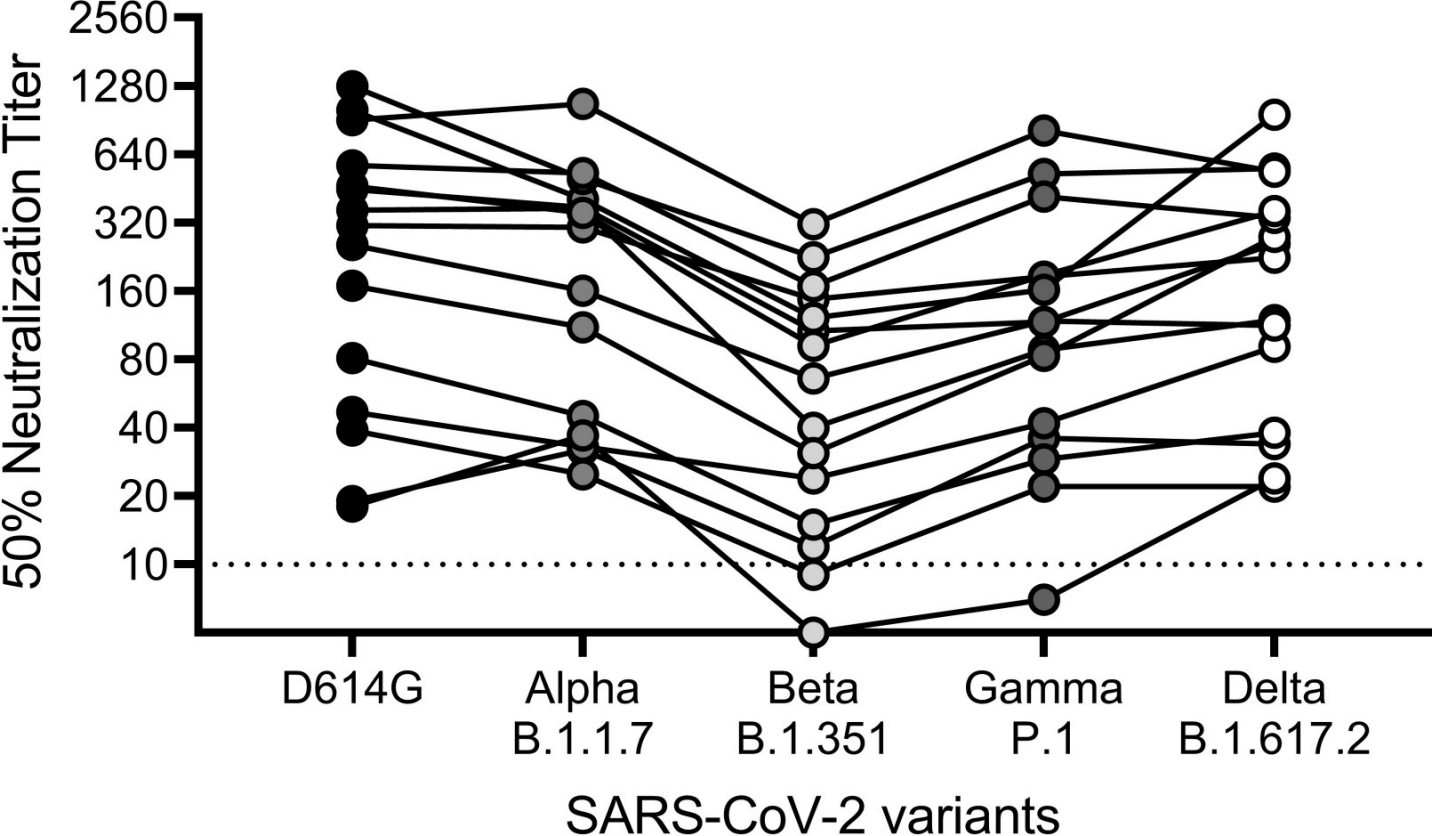
Virus neutralization of SARS-CoV-2 variants of concern. Virus neutralization titers for SARS-CoV-2 convalescent sera against four SARS-CoV-2 variants of concern–Alpha (B.1.1.7), Beta (B.1.351), Gamma (P.1) and Delta (B.1.617.2)–relative to an early pandemic strain (D614G). The comparison between strain variants was done on the same day using a single dilution for each serum sample. ELISA primary antibody: mouse mAb BSM-41414M.

To evaluate the assay performance with different SARS-CoV-2 variants, we determined the virus neutralization of convalescent plasma against four SARS-CoV-2 variants of concern. These include Alpha (B.1.1.7), Beta (B.1.351), Gamma (P.1), and Delta (B.1.617.2). The variants were titrated on Vero E6 cells 96 hours post-inoculation and 300 TCID_50_ virus were used. The variants of concern were tested concurrently with an early pandemic strain using a set of convalescent sera (n = 15) representing the full range from low to high titers. Cross-neutralization was seen for all virus variants. An overall lowering of measured sample titers was observed (Fig 8), when comparing to the early pandemic strain D614G (median titer 283), with median titers of 233 (Alpha [B.1.1.7]; *p* = 0.0398), 53 (Beta [B.1.351]; *p* = <0.0001), 103 (Gamma [P.1]; *p* = 0.0004) and 172 (Delta [B.1.617.2]; *p* = 0.0145).

## Discussion

We developed and evaluated a robust and reliable live-virus assay for detection of neutralizing antibodies against SARS-CoV-2. We provide perspectives with the proposal of two models for a weighed normalization strategy enabling an enhanced inter-assay comparison and unification of results which markedly reduce the degree of variation both within and between assays. Through monitoring performance of a key-component in the assay, suboptimal runs can be adjusted and optimized. Our results show that the impact of assay variation increases with increasing titers. For this reason, we present two different approaches for normalization of results to overcome intra-assay and inter-assay variation with a mild impact on low titered samples and an increasing impact on higher titered samples. Both models reduce the necessity to rerun assays not displaying standard performance, with a massive fine-tuning of results in the higher end. Model 1 is based on the assumption that variation has a linear relation with sample titer (S4 Fig). Model 2 is based on the systematic effect a suboptimal run has on each sample group determined from the low, medium and high titered samples in the precision study. This heteroscedastic relationship between titer and variance, is optimally fitted with a 2^nd^ degree polynomial curve (S4 Fig). Model 1 can easily be adopted to other performing laboratories by determining a nominal value of the used positive control material. We determined a nominal value based on 8 optimal runs within the 15 assay precision study. A more exact estimate of the positive control could be reached by increasing the number of repeats to 20 or more. However, including data from 16 post-precision study runs showed only a moderate change of the mean positive control value (S5 Fig). Where Model 1 is a plug-n-play tool applicable for all laboratories determining the nominal value of their positive control material, Model 2 in contrast is considered laboratory specific, however, with an improved intra-laboratory normalization power. Model 2 is at this stage more theoretical, based on results from a relatively small dataset, accordingly, more intensive knowledge about the influence variation in the assay has on low, medium and high titered samples, would strengthen the model, and hence make the normalization more accurate. To adapt the approach used in Model 2, multiple empirical determinations covering the entire range has to be conducted to generate a model specific for each laboratory, however with a potential to be a tailored powerful normalization strategy. Both Model 1 and Model 2 are our approach to correct for the observed heteroscedasticity; however, as opposed to other models correcting for this phenomena [22], these proposed models simultaneously takes the degree of inaccuracy of the positive control into the calculation. If the positive control is not deviating from the nominal value, results in the entire range are considered accurate and correction is not applied.

Assay performance was assessed by selecting relevant parameters of evaluation. Our studies show a successful performance, with a repeatability (intra-assay variation) and an intermediate precision (inter-assay variation) in the entire range that can be reduced to 15.3% and 16.5%, respectively, when normalization is applied. This meets the acceptance criteria of 25% set for similar neutralization assays by other studies. All replicate results, from the precision studies, fall within three fold of the median titer limit set in similar studies evaluating neutralization assays [23, 24] and within two fold of the mean titer set by others [15]. Using standardized titers, where exact normalized titers are rounded to the closest standard titer, all replicates vary with a maximum of one titer step in the low, the medium and the high titered groups (S1 Table).

We evaluated the assay’s ability to correctly identify SARS-CoV-2 antibody positive and negative samples by analyzing a panel of known positive and negative samples from naturally infected patients, and comparing results to a commercially available SARS-CoV-2 antibody test. The assay detected neutralizing antibodies in 97.6% of samples that were positive for SARS-CoV-2 antibodies in the Wantai Total Antibody ELISA kit and did not detect neutralizing antibodies in any of the samples negative for SARS-CoV-2 antibodies in the same ELISA. The assay furthermore displayed a high level of accuracy with titers similar to the reference laboratory, in a European study, where 12 laboratories in 8 European countries participated in a quality assurance study of convalescent plasma for clinical trials [25]. The assay was specific for serum samples even if run undiluted. EDTA plasma samples run undiluted displayed non-specific virus inhibition which is reduced if diluted 1:5 and insignificant if diluted 1:10. This has previously been proposed to be a result of cellular toxicity of the anticoagulants used for plasma preparation [26].

Though still regarded as the golden standard for the assessment and quantification of functional neutralizing antibodies [27], live-virus microneutralization assays are complex, labor-intensive and time consuming assays. They require highly skilled personnel, BSL3 laboratory facilities for SARS-CoV-2 and a very strict and comprehensive laboratory infrastructure. In addition, assays involving sample titrations have a lower throughput than traditional qualitative assays leaving it more cumbersome to analyze sample populations at a larger scale. Standard immunological assays on the contrary are easy to perform, easy to upscale, less time consuming and does not require BSL3 facilities. Several correlation studies have been performed assessing the applicability of other immunological assays such as ELISA for the use in functional antibody screening [28–31]. Some report a good correlation between ELISA and neutralization assays [28, 30], while others report poor agreement, leaving other serological assays less fit-for-purpose for use in convalescent plasma therapy and other antibody functionality studies [29]. For SARS-CoV-2 variants, the level of spike binding antibodies measured by serological assays do not necessarily reflect the neutralizing antibody levels. Thus, for functional antibody measurements, neutralization assays should be used [32].

Other neutralizing antibody assays like replicating surrogate virus assays, non-replicating pseudovirus assays and non-viral binding assays can equally be performed in a standard BSL2 environment. These types of neutralization assays rely on detecting neutralizing antibodies against fragments of SARS-CoV-2, and not the entire virus particle. In a comparative study of a live-virus neutralization assay, a replicating surrogate virus assay, a non-replicating pseudovirus assay and a SARS-CoV-2-spike RBD binding assay, performed on longitudinal samples, the SARS-CoV-2-spike RBD binding assay failed to detect a decline in titer over a six months period time [33]. These differences most likely arise from the absence of other viral epitopes important for antibody neutralization such as the N-terminal domain of the spike protein, the envelope protein and the membrane proteins. In contrast, live-virus assays detect functional neutralizing antibodies against every part of the live virus particle. This gives live-virus based assays an intuitively better specificity and sensitivity because of its usage of the authentic virus, thereby improving the mimicking of the clinical situation [33, 34]. Pseudovirus neutralization assays remain a useful tool for laboratories lacking BSL3 infrastructure and for viruses difficult to isolate and grow in cell culture. To ensure comparability between live and pseudovirus neutralization assays, universal standard are encouraged since pseudovirus assays tend to measure higher titers with a good correlation but a decreased agreement with other neutralization assays [35]. Pseudovirus neutralization assays tend to over-evaluate titers in samples with low neutralizing activities, but with an improved correlation in the higher titered samples [34].

Design flexibility with a live virus platform furthermore offers the opportunity to exchange the virus isolate relatively easy thereby allowing an assessment of cross-neutralization of other variants of SARS-CoV-2. These studies however, show that clinical isolates differ markedly in the rate of which they replicate in Vero E6 cells. Accordingly, growth kinetics of the clinical isolate has to be considered, taking into consideration that a prolonged infection incubation will diminish ELISA linearity. Studies in the SARS-CoV-2 Cluster 5 variant showed a slower growth of the E gene confirmed by qPCR and concurrent lower level of nucleocapsid protein post 24 hours as also observed for some variants in this study. Thus, the reduced level of nucleocapsid detection is likely a result of slower replication and not lower production of the nucleocapsid protein [36]. We optimized and evaluated the neutralization assay prior to the emergence of the Omicron variant of concern, although the assay is appropriate for this variant as well [37]. Due to the slower endocytic entry pathway used by the Omicron variant, the virus should be titrated at 120 hours and during the neutralization assay, the incubation period adjusted to between 28 and 32 hours to allow for sufficient nucleocapsid protein production.

We believe that this assay could be implemented relatively quickly for laboratories using the similar assay for influenza as described by the WHO, providing the means for a standardized semi-quantitative detection of SARS-CoV-2 neutralizing antibodies. Laboratories performing routine influenza neutralizing antibody surveillance, will have no difficulties in adapting to this assay. No highly specialized equipment is needed. An ELISA reader determining OD values is standard laboratory equipment, giving a uniform readout, which has an advantage over the more subjective visual interpretation in CPE or plaque readings. Concrete data offers the opportunity to normalize results and thereby lowering assay variation and improving standardization of results. In addition, an ELISA readout reduces the total run time of the assay with several days, markedly improving the throughput of the assay in comparison to CPE based live-virus assays or plaque reduction neutralization tests. Moreover, using cultured clinical isolates as antigenic analytes, the assay is empowered by an easily conversion to emerging variants of interest.

## Conclusions

A SARS-CoV-2 live-virus TCID_50_ neutralizing antibody assay was developed, optimized and evaluated using a WHO platform well-known for determining influenza neutralizing antibodies. The assay was tested for specificity, sensitivity, precision, lower level of sample dilution for optimal quantification and linearity. We propose two different models for a weighed normalization of the results providing an enhanced precision and inter-assay result comparison. Model 1 can relatively easy be implemented in other laboratories. The assay is suggested for standard use for comparing SARS-CoV-2 neutralization assay results across reference laboratories.

## Supporting information

S1 Fig Titration of anti-SARS-CoV-2 monoclonal antibodies and appropriate secondary antibodies for the ELISA. (PDF)

S2 Fig Varying incubation times with the acetone-PBS fixative have on the ELISA assay. (PDF)

S3 Fig Virus titration using a SARS-CoV-2 nucleocapsid ELISA or cytopathic effect as read-out for virus positive wells. (PDF)

S4 Fig Titer weight factor curves as a function of titer. (PDF)

S5 Fig Positive control evaluation. (PDF)

S1 Table Precision measurements. (PDF)

S1 Dataset (XLSX)
