# Supplementary material for: Optimization and evaluation of a live virus SARS-CoV-2 neutralization assay
Source: PLoS One. 2022 Jul 28;17(7):e0272298. doi: 10.1371/journal.pone.0272298 (PMC9333216; doi:10.1371/journal.pone.0272298)
Supplement: S1 Fig — (PDF) [file pone.0272298.s001.pdf]

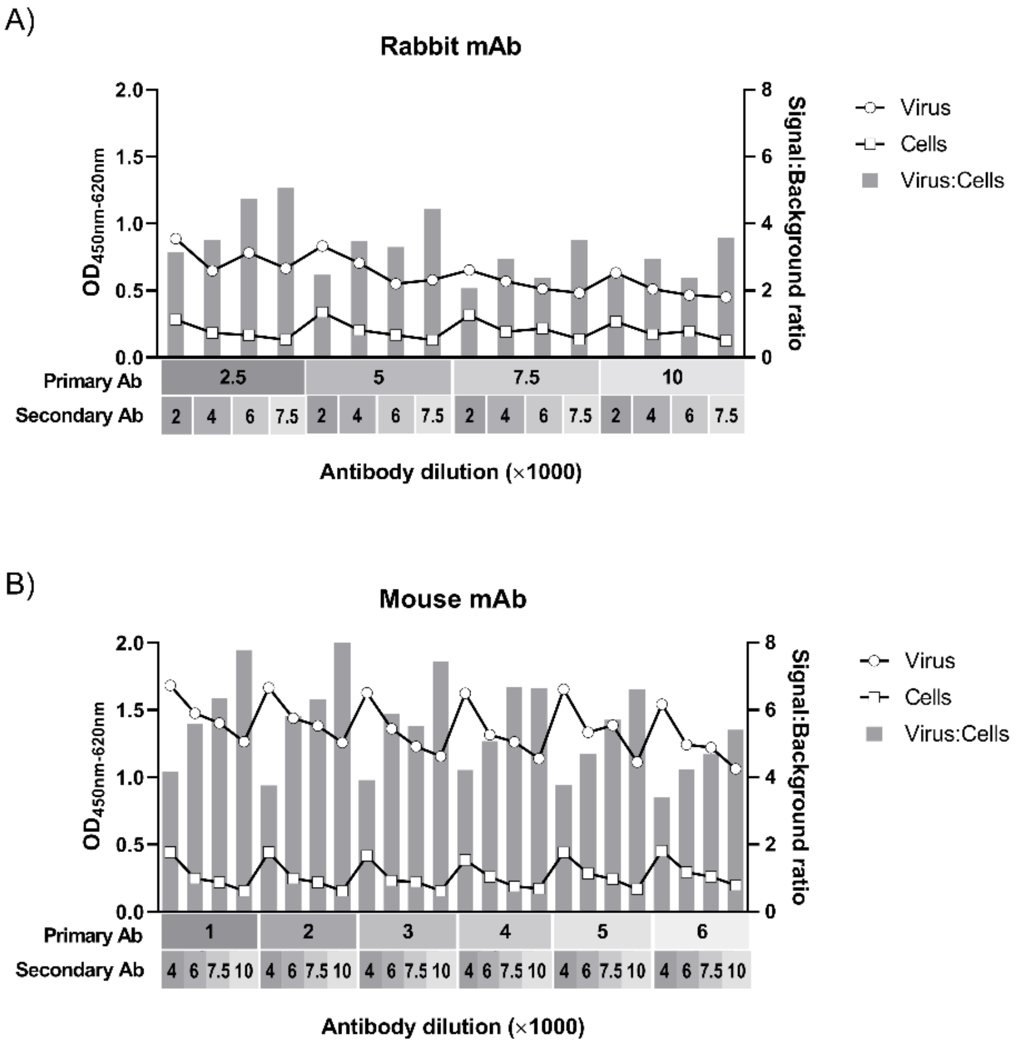

S1 Fig. Titration of anti-SARS-CoV-2 monoclonal antibodies and appropriate secondary antibodies for the ELISA. Duplicate wells of Vero E6 cells infected with 300x TCID50 virus was tested with each dilution for the anti-SARS-CoV-2 monoclonal antibody and the appropriate secondary antibody (circles). The signal in the presence of nucleocapsid protein was compared to the background signal of duplicate mock infected cells on the same assay plate using the same antibody dilutions (squares). Grey bars indicate the signal to background ratio for each dilution. The dilutions used for the primary and secondary antibodies are indicated at the bottom of each graph. A) Rabbit anti-SARS-CoV-2 nucleocapsid protein monoclonal antibody (cat. #40143-R019, Sino Biological, Beijing, China) with secondary antibody mouse anti-rabbit IgG (γ-chain specific) peroxidase-conjugated monoclonal antibody clone RG-96 (cat. #A1949, Sigma Aldrich, Merck, Darmstadt, Germany). B) Mouse anti-SARS-CoV-2 nucleocapsid protein monoclonal antibody clone 7E1B (cat. #BSM-41414M, Bioss Inc, Woburn, MA, USA) with secondary antibody goat anti-mouse IgG peroxidase-conjugated polyclonal antibody (cat. #A16078, Invitrogen, Thermo Fisher).
