# Supplementary material for: Optimization and evaluation of a live virus SARS-CoV-2 neutralization assay
Source: PLoS One. 2022 Jul 28;17(7):e0272298. doi: 10.1371/journal.pone.0272298 (PMC9333216; doi:10.1371/journal.pone.0272298)
Supplement: S2 Fig — (PDF) [file pone.0272298.s002.pdf]

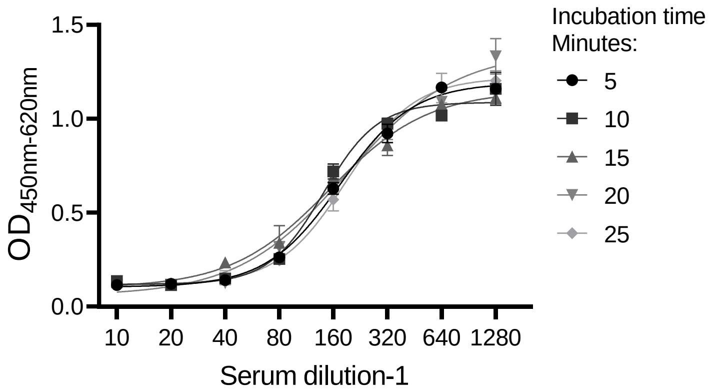

S2 Fig. Varying incubation times with the acetone-PBS fixative have on the ELISA assay. Virus neutralization of a 2-fold serial diluted serum sample was tested using different incubation times for the cells with the cold 80% acetone (v/v) in PBS. All other variables were kept constant. Cells were fixed for 5, 10, 15, 20, or 25 minutes. Each point represents the mean of two replicates and the error bars the range. A 4-parameter logistics regression curve was fitted over the mean data points for each incubation period. ELISA primary antibody: mouse mAb BSM-41414M.
