# Supplementary material for: Optimization and evaluation of a live virus SARS-CoV-2 neutralization assay
Source: PLoS One. 2022 Jul 28;17(7):e0272298. doi: 10.1371/journal.pone.0272298 (PMC9333216; doi:10.1371/journal.pone.0272298)
Supplement: S3 Fig — (PDF) [file pone.0272298.s003.pdf]

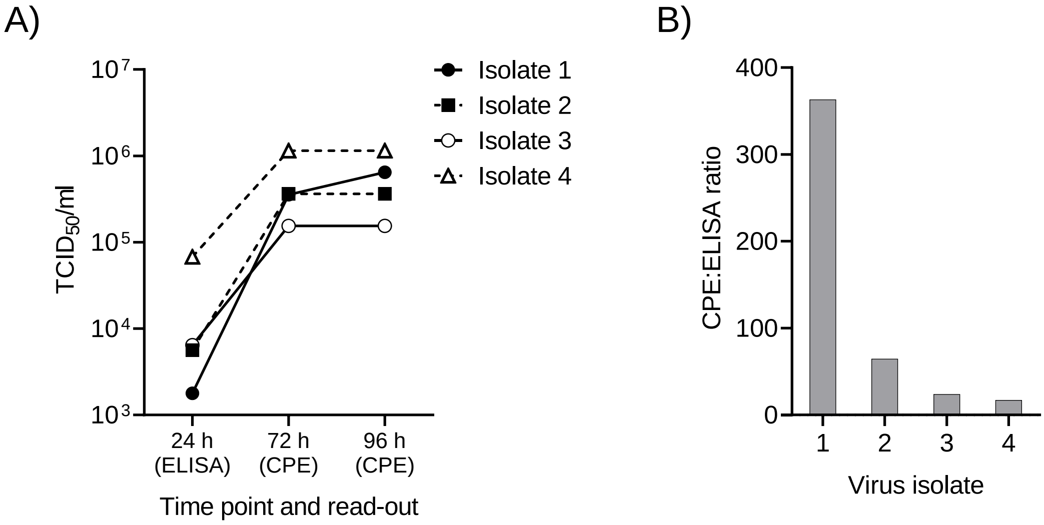

S3 Fig. Virus titration using a SARS-CoV-2 nucleocapsid ELISA or cytopathic effect as read-out for virus positive wells. Four early pandemic SARS-CoV-2 virus isolates were each diluted serially 10^{-1} to $10^{-6.5}$ in a master volume and transferred to two identical cell culture plates seeded with 10000 Vero E6 cells per well the day prior. Virus titrations were performed in quadruplicates. (A) $TCID_{50}$/ml calculated using the Reed-Muench method on the basis of ELISA and CPE read-out after 72 hours and 96 hours. ELISA primary antibody: rabbit mAb 40143-R019. (B) Ratio of calculated $TCID_{50}$/ml from CPE after 96 hours and from ELISA after 24 hours.
