# Supplementary material for: Optimization and evaluation of a live virus SARS-CoV-2 neutralization assay
Source: PLoS One. 2022 Jul 28;17(7):e0272298. doi: 10.1371/journal.pone.0272298 (PMC9333216; doi:10.1371/journal.pone.0272298)
Supplement: S4 Fig — (PDF) [file pone.0272298.s004.pdf]

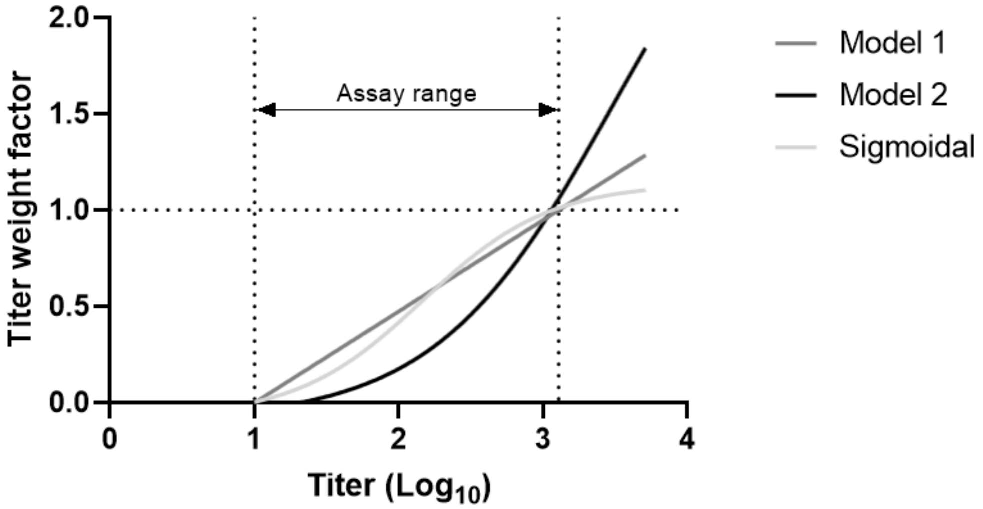

S4 Fig. Titer weight factor curves as a function of titer. Visualization of the range of the titer weight factor segment of Model 1, Model 2 and an experimental sigmoidal test curve. In a standard assay, samples are serial diluted from 1:10 to 1:1280 with a maximum titer weight factor of 1 resulting in a full effect of the positive control correction from fraction 2 in Model 1 and Model 2. Vertical dotted lines represent the assay range in a standard assay. Horizontal line represents the maximum titer weight factor a sample can attain in a standard assay. Test sigmoidal curve displayed is y = -0.1+(1.17-(-0.1))/(1+10^((2.2-x)*0.9)). This curve performs equally well with Model 1 (repeatability: 12.2%, 14.5%, 14.6%; intermediate precision: 16.0%, 24.2%, 14.9% in the low, medium, high group), however as it is not outperforming Model 1 or Model 2, evaluated by the effect on intermediate precision and repeatability, it is not discussed further. It is proposed here as platform for an alternative model with a moderate weight factor in the very high titer region above standard assay range.
