# Supplementary material for: Optimization and evaluation of a live virus SARS-CoV-2 neutralization assay
Source: PLoS One. 2022 Jul 28;17(7):e0272298. doi: 10.1371/journal.pone.0272298 (PMC9333216; doi:10.1371/journal.pone.0272298)
Supplement: S5 Fig — (PDF) [file pone.0272298.s005.pdf]

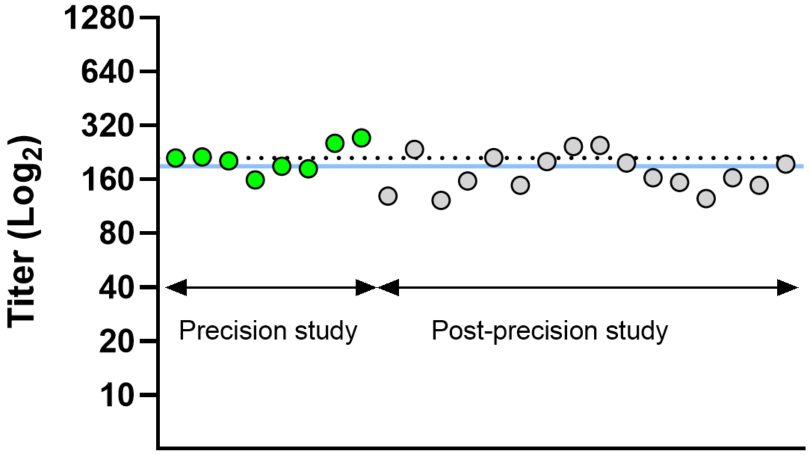

S5 Fig. Positive control evaluation. Titer of the positive control included in each assay, displaying the 8 determinations regarded as optimal and used for the preliminary nominal value calculations from the precision study (green) and 16 post-precision study assays. Dotted line represents nominal value (titer: 211), which is the mean of the 8 optimal runs. Blue line represents the mean of all data points displayed (titer: 189).
