# Supplementary material for: Optimization and evaluation of a live virus SARS-CoV-2 neutralization assay
Source: PLoS One. 2022 Jul 28;17(7):e0272298. doi: 10.1371/journal.pone.0272298 (PMC9333216; doi:10.1371/journal.pone.0272298)
Supplement: S1 Table — (PDF) [file pone.0272298.s006.pdf]

S1 Table. Precision measurements.

			Exact titer					Categorical titer				
			Intra-Assay Replicate									
			# 1	# 2	# 3	# 4	# 5	# 1	# 2	# 3	# 4	# 5
Calculated titer (Raw data)	Low titer	Day 1	53	47	59	39	49	40	40	40	20	40
		Day 2	68	85	64	72	63	40	80	40	80	40
		Day 3	59	62	55	45	53	40	40	40	40	40
		Day 4	42	42	47	56	46	40	40	40	40	40
		Day 5	50	56	44	51	45	40	40	40	40	40
	Medium titer	Day 1	217	189	227	192	158	160	160	160	160	160
		Day 2	285	254	316	311	233	160	160	160	320	160
		Day 3	160	208	196	172	144	160	160	160	160	80
		Day 4	143	199	152	173	193	80	160	80	80	160
		Day 5	164	170	190	191	135	80	160	160	160	80
	High titer	Day 1	680	670	729	647	610	640	640	640	320	320
		Day 2	510	580	637	581	530	320	320	320	320	320
		Day 3	1685	1397	1808	2356	1892	1280	1280	1280	1280	1280
		Day 4	701	605	635	614	644	640	320	320	320	640
		Day 5	1252	1844	1627	2171	2310	640	1280	1280	2560	1280
			**Exact titer**					**Standardized titer**				
Calculated titer (Normalized model 1)	Low titer	Day 1	53	47	59	39	49	40	40	40	40	40
		Day 2	60	73	56	63	56	40	80	40	80	40
		Day 3	58	62	55	45	53	40	80	40	40	40
		Day 4	42	43	47	57	46	40	40	40	40	40
		Day 5	57	64	49	58	51	40	80	40	40	40
	Medium titer	Day 1	262	226	276	230	185	320	160	320	160	160
		Day 2	179	163	194	191	153	160	160	160	160	160
		Day 3	170	223	210	183	152	160	160	160	160	160
		Day 4	112	149	119	132	145	160	160	160	160	160
		Day 5	205	213	243	244	165	160	160	320	320	160
	High titer	Day 1	1202	1180	1310	1131	1053	1280	1280	1280	1280	1280
		Day 2	1186	1410	1603	1414	1247	1280	1280	1280	1280	1280
		Day 3	1287	1084	1372	1745	1430	1280	1280	1280	1280	1280
		Day 4	1380	1146	1218	1167	1241	1280	1280	1280	1280	1280
		Day 5	905	1274	1141	1471	1553	640	1280	1280	1280	1280
Calculated titer (Normalized model 2)	Low Titer	Day 1	53	47	59	39	49	40	40	40	40	40
		Day 2	66	81	62	70	61	80	80	80	80	80
		Day 3	59	62	55	45	53	40	80	40	40	40
		Day 4	42	42	47	56	46	40	40	40	40	40
		Day 5	51	58	45	52	46	40	40	40	40	40
	Medium Titer	Day 1	241	208	254	211	170	320	160	320	160	160
		Day 2	212	194	228	225	182	160	160	160	160	160
		Day 3	164	216	204	178	147	160	160	160	160	160
		Day 4	128	170	136	151	166	160	160	160	160	160
		Day 5	183	190	216	218	147	160	160	160	160	160
	High Titer	Day 1	1122	1099	1235	1049	970	1280	1280	1280	1280	1280
		Day 2	1002	1218	1411	1222	1060	1280	1280	1280	1280	1280
		Day 3	1242	1063	1316	1631	1366	1280	1280	1280	1280	1280
		Day 4	1279	1039	1112	1061	1136	1280	1280	1280	1280	1280
		Day 5	890	1202	1093	1359	1423	640	1280	1280	1280	1280

Exact titer = titer calculated according to a four-parameter logistic regression curve fit from non-normalized (raw data) or normalized OD-values; Categorical titer = the highest serum dilution positive for neutralization activity determined from non-normalized OD-values (raw data); Standardized titer = the closest standard dilution factor on a $\log_2$ scale starting from a 1:10 dilution calculated from normalized OD-values.
